# A Laboratory Guide on Urinalysis and Toxicology

**Published:** 1886-12

**Authors:** 


A Laboratory Guide on Urinalysis and Toxicology. By R. A. Withans, A. M., M. D. Wm. Wood & Co.

The author of this little work has no preface in which he proves that his book will "fill a long-felt want." There is no intimation of how much time the "busy practitioner" will save by keeping a copy always at hand. In the absence of such introductory remarks, we judge from the title that the book is intended, not for the general practitioner, but solely for the chemists' laboratory. It is a laboratory guide, and as such presents many commendable features. There are several objections to it, however, which must impair its usefulness, even with students in the laboratory. In the first place, the reagents used in the various tests are spoken of always by their chemical symbols; for instance, aq. ammonia appears as $NH_4HO$, etc. Medical students, as a rule, know very little about chemistry, and this use of chemical symbols complicates matters considerably. Another more serious objection is that there is no mention whatever of the various diseases which produce abnormal conditions of the urine, so that after the student finds that a specimen of urine contains hyaline casts, or uric acid crystals, he must refer to his text-books in order to learn what diseases are attended with the appearance of such ingredients in the urine.

The latter half of the book is devoted to the "Detection of Poisons." The tests for volatile, mineral and organic poisons are concisely stated. The directions for conducting the tests are so explicitly given that even a beginner could perform many of them.

To the practical chemist it will doubtless prove a very valuaable and convenient guide. There are other manuals, however, on urinary analysis which meet the wants of the student and the practitioner better than the work of Dr. Withans. The name of the publishers is a guarantee of its typographical excellence.

